# Epilepsy in Tubulinopathy: Personal Series and Literature Review

**DOI:** 10.3390/cells8070669

**Published:** 2019-07-02

**Authors:** Romina Romaniello, Claudio Zucca, Filippo Arrigoni, Paolo Bonanni, Elena Panzeri, Maria T. Bassi, Renato Borgatti

**Affiliations:** 1 Neuropsychiatry and Neurorehabilitation Unit, Scientific Institute, IRCCS Eugenio Medea, Bosisio Parini, 23842 Lecco, Italy; 2 Clinical Neurophysiology Unit, Scientific Institute, IRCCS Eugenio Medea, Bosisio Parini, 23842 Lecco, Italy; 3 Neuroimaging Lab, Scientific Institute, IRCCS Eugenio Medea, Bosisio Parini, 23842 Lecco, Italy; 4 Epilepsy and Clinical Neurophysiology Unit, Scientific Institute, IRCCS Eugenio Medea, Conegliano, 31015 Treviso, Italy; 5 Laboratory of Molecular Biology, Scientific Institute, IRCCS Eugenio Medea, Bosisio Parini, 23842 Lecco, Italy

**Keywords:** tubulin genes, epilepsy, malformations cortical development, EEG, *TUBA1A*, *TUBB2B*, *TUBB3*

## Abstract

Mutations in tubulin genes are responsible for a large spectrum of brain malformations secondary to abnormal neuronal migration, organization, differentiation and axon guidance and maintenance. Motor impairment, intellectual disability and epilepsy are the main clinical symptoms. In the present study 15 patients from a personal cohort and 75 from 21 published studies carrying mutations in *TUBA1A*, *TUBB2B* and *TUBB3* tubulin genes were evaluated with the aim to define a clinical and electrophysiological associated pattern. Epilepsy shows a wide range of severity without a specific pattern. Mutations in *TUBA1A* (60%) and *TUBB2B* (74%) and *TUBB3* (25%) genes are associated with epilepsy. The accurate analysis of the Electroencephalogram (EEG) pattern in wakefulness and sleep in our series allows us to detect significant abnormalities of the background activity in 100% of patients. The involvement of white matter and of the inter-hemispheric connection structures typically observed in tubulinopathies is evidenced by the high percentage of asynchronisms in the organization of sleep activity recorded. In addition to asymmetries of the background activity, excess of slowing, low amplitude and Magnetic Resonance (MR) imaging confirm the presence of extensive brain malformations involving subcortical and midline structures. In conclusion, epilepsy in tubulinopathies when present has a favorable evolution over time suggesting a not particularly aggressive therapeutic approach.

## 1. Introduction 

In the last two decades, more evidence has been provided about the role of genes in determining epilepsy [1,2,3,4,5]. Among these genes, some play a role in neuronal membrane electrical stabilization [1], while others are involved in or regulate neuronal proliferation, migration and postmigrational cortical organization during fetal brain development. Mutations in this last group of genes lead to major structural cortical abnormalities (malformations of cortical development, MCDs) which are associated with specific neuroradiological patterns [2,3]. In the last years, the improvement and availability of new molecular genetics and neuroradiology techniques allowed a great increase of studies on the genes involved in the processes of cerebral cortical development, and more than 100 genes have been reported to date [2,4,5,6,7]. A key role in determining MCDs is played by microtubules (MTs), proteins that provide structures and forces needed by neurons to migrate and to develop axonal and dendritic processes [8]. Tubulins represent the major constituents of microtubules; they are dimeric proteins consisting of two closely α and β related subunits [9] encoded by tubulin genes (i.e., *TUBA1A, TUBB2B, TUBB3, TUBB4A, TUBB2A*, *TUBB*, *TUB8A*). Tubulin proteins play a key role in several cellular processes crucial for a proper cortical development during neuronal proliferation, migration, differentiation, cortical laminar organization and axonal guidance of the radial glia (axon outgrowth and maintenance) [10,11] Mutations in the α- and β- tubulin genes, related to the functional area of the protein involved, lead to a complex and wide spectrum of cerebral malformations defined “Tubulinopathies” [12,13,14]. Typically, affected patients show complex malformations of cortical development (e.g., polymicrogyria, lissencephaly, pachygyria, subcortical band heterotopia, schizencephaly etc.), basal ganglia dysmorphisms (frequently characterized by an agenesis or thinning of the anterior limb of internal capsule - ALIC) and commissural anomalies (different combinations of anterior commissure and corpus callosum agenesis or dysmorphisms) [15,16,17,18,19,20,21,22,23,24,25,26,27,28,29,30,31,32,33,34,35,36,37,38,39]. The abnormalities of the posterior fossa have been systematically described only in the last years. The most frequent findings are cortical cerebellar dysplasia, brainstem asymmetries and brainstem clefts [40,41,42].

The present study describes in detail the tubulin genes family and its role in brain development and in epileptogenic mechanisms. EEG and clinical data of six epileptic patients from a cohort of 15 patients carrying mutations in *TUBA1A*, *TUBB2B* and *TUBB3* tubulin genes are analyzed as well as a literature review on the role of these genes and epilepsy is reported with the aim to define a specific associated epileptic pattern.

## 2. Material and Methods

Fifteen patients (seven males, age range 4–56 years) carrying mutations in *TUBA1A*, *TUBB2B* and *TUBB3* genes were enrolled in the study from a personal cohort of patients. Blood samples were obtained from the patients and available parents and genomic DNA was extracted using conventional strategies. All patients were screened using a targeted next generation sequencing approach with a gene panel including 149 genes known to be involved in MCD and Corpus Callosum Agenesis (CCA) (the gene list is reported in Supplementary file). To exclude variants or mutations in epilepsy genes, all patients were also screened by using a NGS gene panel including 170 genes known to be mutated in epileptic encephalopathies and other genetic forms of epilepsy (the gene list is reported in Supplementary file). The targeted regions were designed to include coding exons with intronic 50 bp flanking sites and 3′ and 5′ untranslated regions (UTRs) by using the SureDesign system (Agilent Technologies, Santa Clara, CA, USA). The sequencing libraries were prepared from genomic DNA by using a Sure Select enrichment system (Agilent Technologies). Targeted libraries were run on MiSeq/NextSeq platform according to the manufacturer’s instructions (Illumina, San Diego, CA, USA). ANNOVAR was used for annotation against the RefSeq database and the Single Nucleotide Polymorphism databases. The filtering strategy we applied led us to select only variants located in the coding regions including the splice site (synonymous variants were excluded), variants that exhibited a minor allele frequency of less than 1% or were not present in variant databases including those of the 1000 Genomes Project, the Exome Aggregation Consortium (ExAC) and the NHLBI exome sequencing project ESP6500. On average, 98.66% and 99.3% of bases were covered by at least 10 and 20 sequence reads, respectively. Sanger Sequencing was used to confirm all the identified variants. For each patient, the identified mutation was tested in both parents when available to assess whether it occurred de novo or was inherited from one of the parents. Mutations nomenclature is based on RefSeq NM_006009.3 (*TUBA1A*), NM_178012.4 (*TUBB2B*) and NM_6086.2 (*TUBB3*) considering the A of the ATG as nucleotide 1 and follows the guidelines of the Human Genome Variation Society. 

Novel variants were tested against different databases: 1000 genomes, Exome Variant Sever, dbSNP, HGMD (Human Gene Mutation Database) and ExAC (Exome Aggregation Consortium) databases. To confirm the pathogenicity of the mutations, we used web-based prediction programs: MutPred (http://mutpred.mutdb.org), PolyPhen-2 (http//genetics.bwh.harvard.edu/pph2), PROVEAN (http://provean.jcvi.org/), Mutation taster (http://mutationtaster.org) and SIFT (http://sift.jcvi.org/www/SIFT_seq_submit2.html).

All patients had a brain MRI scan performed either on a 1.5 T or 3 T MR scanner for clinical indication using the local departmental protocol. For each patient, MRI included a three-dimensional (3D) T1-weighted sequence and two-dimensional (2D)-Turbo Spin Echo T2-weighted sequences on at least two orthogonal planes. All the exams were reviewed by an experienced neuroradiologist who classified the neuroimaging finding of each patient.

Clinical data on epilepsy symptomatology and electrophysiological records of all patients were collected in detail. Particularly were identified and classified: age of seizure onset, type of seizure, epileptic syndrome and the response to anti-epileptic treatment. All the EEG recordings of the patients included in the study were reviewed and classified, according to the characteristics of background activity during wakefulness and during sleep and the presence and the characteristics of slow and epileptiform abnormalities.

A MEDLINE search on PubMed and OMIM Programs using [Tubulin gene mutations in human brain] and [epilepsy] as inclusion criteria yielded 21 studies published between from 2007 January to March 2019.

The Ethics Committee of the E. Medea Scientific Institute approved the study. A written informed consent was obtained from all participating families.

## 3. Results

Clinical and EEG data: six out of 15 patients presented epileptic seizures (40%) (three with *TUBA1A* gene mutation and three with *TUBB2B* gene mutation; none *TUBB3* carrying gene mutation showed epilepsy). In the affected patients, epilepsy started as epileptic encephalopathy (more often a West syndrome). Subsequently the syndrome evolved towards a focal epilepsy that in the vast majority of cases is well controlled by drug therapy. The most used drug was Sodium Valproate (see Table 1). A close correlation between the presence of MCDs and epilepsy can be observed. All the epileptics subjects but one (5/6; 83%) showed MCD at brain MRI, while in 5/9 (55.5%) non-epileptics patients an MCD was absent.

**Figure 1:**
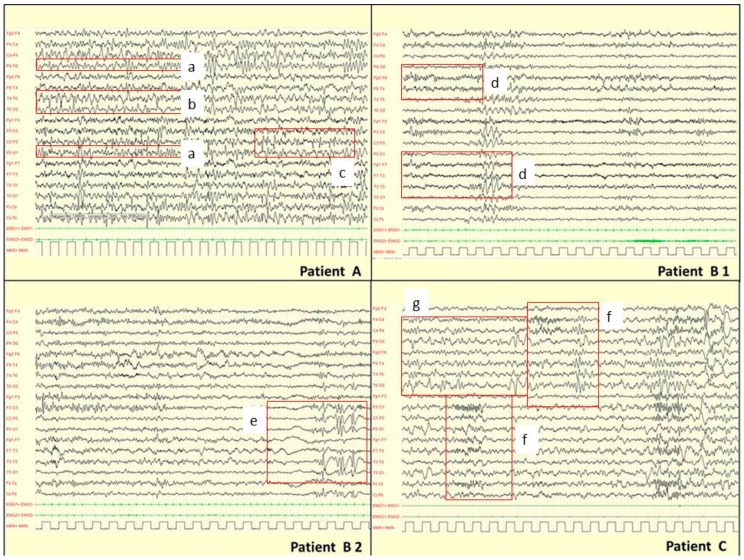
EEG findings in *TUBA1A*, *TUBB2* and *TUBB3* genes mutations. Patient A [P89815] (33 months) has a mutation in *TUBA1A* gene and at MRI multifocal PMG. During drowsiness: asymmetric background activity (square: a). Paroxysmal slow abnormalities over right posterior regions (square: b). Asynchronous epileptiform abnormalities over left central areas (square: c). Patient B [P76712] (8 years) has a mutation in *TUBB2B* gene. B1 EEG during wakefulness: asymmetry of the background activity and paroxysmal slow abnormalities over left centro-temporal areas (square: d). B2 EEG during drowsiness: epileptiform abnormalities over left centro-temporal areas (square e). Patient’s MRI showed a schizencephaly in left centro-temporal areas consistent to EEG abnormalities. Patient C [P105814] (15 months) has a mutation in *TUBB3* gene. EEG during 2-3 non-REM sleep: asymmetric and asynchronous background activity (square f). Diphasic slow abnormalities over right parietal and occipital areas (square g). In this patient, no epileptiform abnormalities were recorded according with the absence of MCDs on MRI.

EEG data were collected in 12 out of 15 patients, and 45 polygraphic recordings of EEG have been analyzed; in 10 patients, several recordings during sleep were also available. Background activity, slow and epileptiform abnormalities collected both during wake and sleep EEG recordings are shown in Table 1. The background activity is significantly abnormal in 100% of patients. Slow and epileptiform recorded anomalies are strictly correlated with the localization of the cortical malformations revealed by MRI (see Figure 1). A high percentage of asynchronisms in the organization of sleep activity was detected. Non-epileptic myoclonus over the surface EMG districts (usually deltoid muscle of both sides) was documented. In one case, paroxysmal EEG abnormalities are evoked by intermittent light stimulation.

Genetic data: 13 variants were identified in *TUBA1A* (8), *TUBB2B* (3), *TUBB3* (4) genes in the 15 patients. Ten variants had been previously reported [41], while three variants are novel (one in *TUBA1A* and two in *TUBB3* genes). In nine families, segregation has been tested: all variants resulted to be de novo apart from *TUBA1A* p.(S54N), which was inherited by the two twin sisters from their affected father (family P113708) [41]. The genetic screening performed for all patients with both the targeted gene panel for MCD genes and a targeted panel for epilepsy genes (see Supplementary file) did not detect any additional mutations.

**Figure 2:**
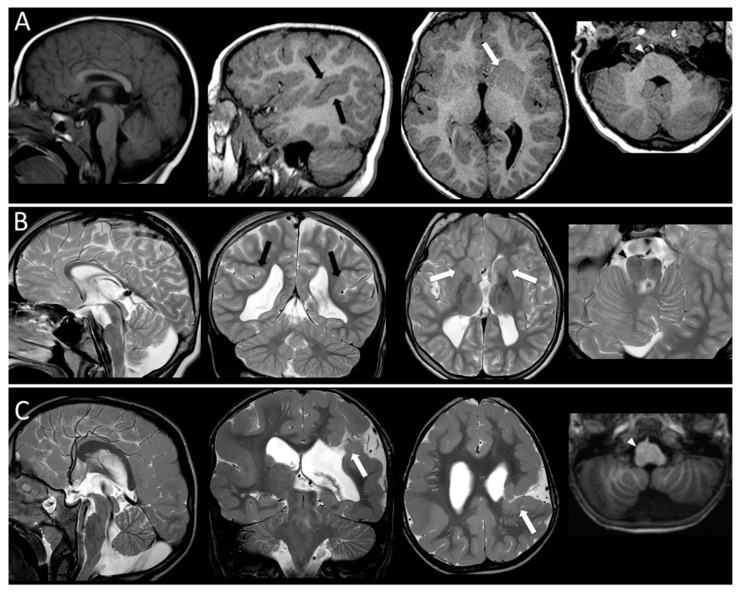
MR imaging findings in three patients. Patient A [P109418] has a mutation in *TUBB3* gene. T1-weighted images show a thin and short corpus callosum, perisylvian polymicrogyria (black arrow), a severe thinning/agenesis of the ALIC (white arrow) and brainstem asymmetry (arrowhead pointing at enlarged pons). Patient B [P45617] carries a mutation in *TUBA1A* gene. Thin corpus callosum, dysgyric cortex in perisylvian areas (black arrows), bilaterally thinned ALIC (white arrows) and pons asymmetry (arrowhead pointing at enlarged pons) are shown on T2-weighted images Patient C [P76712] carries a mutation in *TUBB2B* gene. He has a malformed corpus callosum and brainstem, with a thickened ponto-medullary junction and brainstem asymmetry (arrowhead pointing at enlarged medulla). He has also a complex cortical malformation with diffuse bilateral polymicrogyria and schizencephaly in the left hemisphere (arrows).

Neuroradiological data: mutated patients with epilepsy showed multifocal polymicrogyria (1), generalized polymicrogyria and schizencephaly (2), simplified gyral pattern and linear subcortical heterotopia (1), dysgiria (1) and no MCD in one case. Mutated patients with no epilepsy showed perisylvian polymicrogyria in four cases and normal cortex in five cases. The imaging findings of two patients are reported as example of MCDs and other brain malformations affecting patients with mutations in tubulin genes (see Figure 2). 

**Figure 3:**
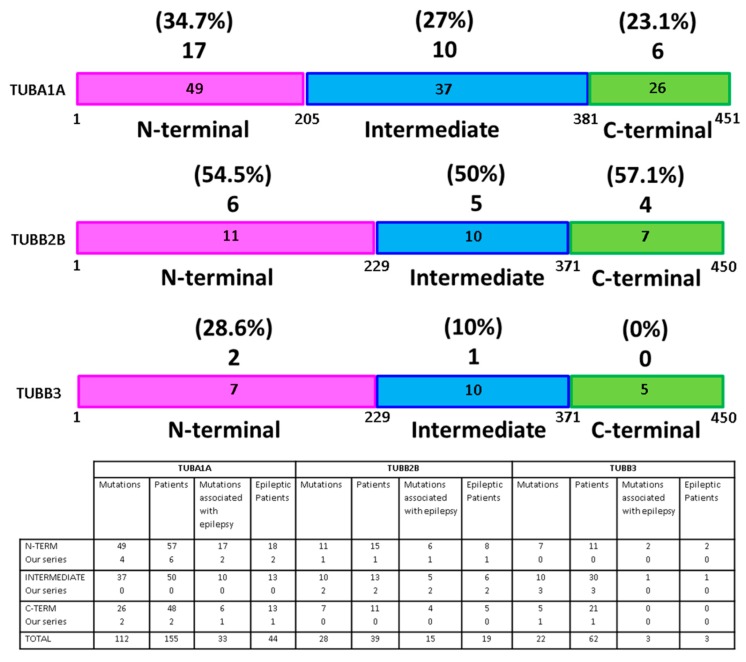
Schematic representations of the functional domains of *TUBA1A, TUBB2B* and *TUBB3*. The numbers in the boxes show how many mutations are described for each tubulin domain by distinguishing literature and our series, while above the protein schemes are reported the number of mutations associated with epilepsy and the corresponding percentage.

Literature data: Figure 3 summarizes results derived from literature review. The number of mutations for each gene and domain and the frequency and characteristics of epileptic features associated are reported. Among patients described in literature carrying mutations in *TUBA1A* gene, epilepsy was reported in 28% (44/155), while it was described in 49% (19/39) of *TUBB2B* and in 5% (3/62) of *TUBB3* mutated patients. Incidence of mutations associated with epilepsy is not significantly different among the tubulin domains for *TUBA1A* and *TUBB2B* genes, while in the *TUBB3* gene, the C-domain does not show mutations related to epileptic features. It is to be noted that the mutations described in this domain do not correlate with MCD phenotype, but with CFEOM (Congenital Fibrosis of the Extraocular Muscles). The seizure onset had a range between birth to 3 years of age. The most frequent seizures types were focal (39% *TUBA1A*; 47% *TUBB2B*) and spasms (26% *TUBA1A*; 33% *TUBB2B*). Absence and myoclonic seizures were reported in 6% (*TUBA1A*) and 7% (*TUBB2B*), respectively. Tonic-clonic seizures were described in 18% (*TUBA1A*) and 13% (*TUBB2B*), while tonic seizures in 8% (*TUBA1A*) and 13% (*TUBB2B*). Only in patients carrying *TUBA1A* gene mutations, focal status epilepticus was observed. Regarding *TUBB3* gene mutations, only febrile seizures were described. Seizure control was achieved in 33% of cases carrying *TUBA1A* gene mutations and in 72% of *TUBB2B* mutated patients. The most used drugs were: Sodium Valproate, Phenobarbital, Carbamazepine, Levetiracetam, Lamotrigine. Refractory seizures were reported only in *TUBA1A* gene mutations (56%) (data summarized in supplementary material Tables S1 and S2).

## 4. Discussion 

The role of α- and β-tubulin genes mutations in determining epilepsy is complex, depending on the way they alter the dynamic properties and functions of microtubules. The main role in causing epilepsy is played by mutations that disrupt stability of MTs, which are primarily involved in neuronal migration and organization, while perturbations of the microtubule dynamic, which have a crucial function in the axonal growth and guidance, are less frequently associated with epileptic phenotypes [43,44,45].

Tubulin genes play a key role in several pathways of cortical development such as other genes encoding microtubule-related proteins (i.e., Lissencephaly 1, Doublecortin, Filamin A or Reelin genes) and when mutated, may cause specific brain malformations strictly associated with epilepsy. Nevertheless, while each of these last genes is correlated with a typical epileptic phenotype (concerning onset, frequency and type of seizures and response to therapy), no specific epileptic pattern was found in tubulinopathies. Despite the complexity of the cerebral malformations and the severity of intellectual disabilities described, epilepsy does not seem to be one of the most serious features of the tubulin related clinical picture. When present, it is described with a wide range of severity (from a mild clinical presentation and spontaneous regression in most of patients, to intractable seizures in rare cases), without a specific clinical pattern [46,47,48]. An explanation for the scanty epileptogenicity of MCDs in tubulinopathies compared to MCDs due to other genes mutations may be the coexisting involvement of basal ganglia and cerebellum [23,28]. In fact literature data [49] support the presence of a “filtering effect” of the basal ganglia which contrasts the spread of ictal epileptiform activity. Other evidences suggest that the cerebellum exerts an inhibitory effect over epileptiform discharges; animal and human studies demonstrated that cerebellar stimulation reduces intensity or shortens seizure [50]. 

Among the α- and β-tubulin genes, *TUBA1A* (28% of the cases) and *TUBB2B* gene (49%) are those more frequently associated with epilepsy. On the contrary, mutations in the *TUBB3* gene cause epilepsy only in 5% of cases. These findings are in line with our personal series, in which only *TUBA1A* and *TUBB2B* mutated patients showed epileptic seizures. Looking at the several genes mutations reported in literature (see Tables S1 and S2) it is evident that there are not significant recurring mutations in *TUBA1A* and in *TUBB2B* genes. On the contrary, in *TUBB3* the mutation E410K is described in many families (13), but all are affected by an ocular motor disorder and no one is epileptic. Analyzing the distribution of mutations along different domains of each gene, none appears to be more frequently involved. Mutations associated with epileptic features are in *TUBB2B* gene equally distributed, while the N-terminal domain is more involved in *TUBA1A* and in *TUBB3* genes. In this last gene, none of the five detected mutations (found in 21 patients) is associated with epilepsy. Intriguingly, in these patients MCDs are also absent. It is possible that this non-uniform distribution of mutations causing epilepsy among the three different genes domain can be attributed to their effect on tertiary structure and to the role played in the mechanisms underlying MCDs. Recently [51] a Tubg1^Y92C/+^ mouse model was developed to investigate the consequence of some *TUBG1* human mutation in MCDs. Cortical and hippocampal neuroanatomical anomalies were found associated with an increased epileptic cortical activities. Histological examinations in *TUBA1A* mutated brains revealed structural abnormalities of the cortex, a fractured pyramidal layer of the hippocampus, and defects attributed to impairment of neuronal migration mechanisms, similar to the mouse models of lissencephaly (*LIS1*, *DCX*, *RELN*) [51,52,53,54]. *TUBB2B* gene mutations influence the tubulin heterodimer folding and their incorporation into microtubules, thus leading to combinations of impairment in neuronal migration and radial glia dysfunction. Regarding the *TUBB3* gene, this is involved primarily in axon guidance mechanisms scantly associated with MCDs and, consequently, with low levels of epileptogenicity. Nevertheless in a recent study, a role of *TUBB3* in regulating epileptic seizures via GABA-A receptor-mediated synaptic transmission is shown, explaining the rare epileptic cases described *TUBB3* mutated patients [55,56,57,58,59,60].

Concerning epileptic clinical manifestation, West syndrome is the most frequent clinical phenotype at onset in patients carrying *TUBA1A* and *TUBB2B* gene mutations. In spite of the severity of this epileptic syndrome (an early epileptic encephalopathy), the response to therapy and the clinical evolution, as described in literature and documented also in our series, are favorable in most cases. Usually, in 70–80% of patients with brain malformations a severe symptomatic epilepsy syndrome is observed [61]. In at least 15% of the cases, these epileptic syndromes are characterized by a high frequency of seizures that are refractory to pharmacological treatment [62]. Despite the relatively small number of patients, it is noteworthy that, in our cohort epilepsy affected only six out 15 of cases, and seizures were refractory to treatment only in one patient. A complete clinical and EEG follow-up of our patients documented the evolution from an epileptic encephalopathy towards a focal epilepsy that, in the vast majority of cases, was well controlled by drug therapy. Five out six of the epileptic patients were seizure free at last control. This data is particularly relevant comparing to the extensions of the referred malformations and the consequent presence of severe neurological and neuropsychological impairments, that are typical risk factors linked to drug-resistant epilepsies. Detailed neurophysiological studies on brain electric activity in tubulinopathies are lacking, but the accurate analysis of the EEG pattern in wakefulness and sleep in our series allows us to detect significant abnormalities of the background activity in 100% of patients. The finding of abnormal bursts of fast activity can be expected in tubulinopathies in relation to the possibility of MCDs but frequently were also detected asymmetries of the activity, excess of slowing, low amplitude that are related to the presence of extensive brain malformations involving other structures beyond the cortex in particular subcortical and midline structures. Moreover, the involvement of white matter and of the inter-hemispheric connection structures is also evidenced by the high percentage of asynchronisms in the organization of sleep activity recorded by EEG in our series [63,64]. In addition, the polygraphic EEG recording allows to detect, in some cases, non-epileptic myoclonus that can be indicative of cerebellum cortical involvement as shown on MRI [41].

In conclusion, epilepsy in tubulinopathies when present has a less unfavorable trend compared with other forms of symptomatic epilepsies in childhood suggesting that, even in presence of a severe epileptic encephalopathy such as West syndrome at onset, no particularly aggressive therapeutic approach can be adopted. Thus, in these patients, early cognitive and behavioral rehabilitation interventions must be carried out in order to allow a better recovery and prognosis. 

## Figures and Tables

**Table 1:** Electrophysiological findings of our series.

	Gender, Age	Epileptic Syndrome	Seizure Type	Age at Onset	Awake EEG BA	Awake EEG EA	Sleep EEG BA	Sleep EEG EA	Response to AEDs	MCDs on MRI	Genetic Findings
**EPILEPTIC PATIENTS**											
**TUBA1A**											
P45617	F, 13 yrs 3 mo	Focal symptomatic epilepsy	Spasms, FS	18 mo, 3 yrs	Irregular Low voltage	0	Irregular BFA Asynchronous	SA EA Right TO	Controlled (ACTH; VPA)	Perisylvian dysgyria	c.466C > G (p.R156G)
P89815 ^§^	F, 3 yrs 3 mo	Focal symptomatic epilepsy	FS	21 days	Irregular Asym Slowing BFA	SA paroxysmal Rght CO	Irregular Asym Asynchronous	SA EA Right CO Left CT asynchronous	Controlled (LEV)	PMG-multi	c.4C > A (p.R2S)
17656 ^§^	M, 13 yrs 4 mo	Focal symptomatic epilepsy	Myoclonic, focal SE, SF	3 mo	Irregular Slowing	0	Irregular Asym	SA paroxysmal -EA Left C PO	Controlled (VPA, ETS)	No MCD	c.1169G > A (p.R390H)
**TUBB2B**											
P76712 ^§^	M, 10 yrs 9 mo	Focal symptomatic epilepsy	Spasms, FS	18 mo	Irregular Asym BFA	SA Left CT	Irregular Asym BFA	SA EA Left CT and diffuse	Partially controlled (VPA)	Generalized PMG + SCH	c.1060T > C (p.C354R) *de novo*
P38408 ^§^	F, 16 yrs 8 mo	Focal symptomatic epilepsy	Spasm, FS	5 mo	Irregular Asym Slowing BFA	0	Irregular Asym BFA NEM	SA EA Right C	Controlled (VPA, LTG)	Generalized PMG + SCH	c.419G > C (p.G140A) *de novo*
P78511 ^§^	F, 39 yrs	Focal symptomatic epilepsy	Spasms, FS	7 mo	Irregular Slow voltage BFA	SA paroxysmal bilateral diffuse positive ILS	NA	NA	Controlled (ACTH, PB, CBZ)	Symp_Gyr, periv heterotopia, subcortical linear heterotopia, small temporal lobes	c.1080_1084delCCTGAinsACATCTTC [p.L361_K362delinsHLQ) *de novo*
**NON EPILEPTIC PATIENTS**											
**TUBA1A**											
P78411 ^§^	M, 7 yrs 6 mo	no	no	/	Irregular Asym Slowing BFA	no	Irregular Asym BFA Asynchronous	SA C bilateral Left predominance	/	No MCD	c.175G > A (p.G59S) *de novo*
P113708 ^§^ I-3	M, 56 yrs 4 mo	no	no	/	NA		NA	NA	/	Perisylvian-PMG	c.161G > A (p.S54N)
P113708 ^§^ II-1	F, 25 yrs 4 mo	no	no	/	Irregular Slowing Low voltage	0	NA	NA	/	Perisylvian-PMG	c.161G > A (p.S54N)
P113708 ^§^ II-2	F, 25 yrs 4 mo	no	no	/	Irregular Slowing Low voltage BFA	0	NA	NA	/	Perisylvian-PMG	c.161G > A (p.S54N)
P76111 ^§^	F, 12 yrs 3 mo	no	no	/	Irregular Asym. Slowing BFA Low voltage	0	Irregular Asym Asynchronous	SA bilateral diffuse	/	No MCD	c.1160C > T (p.A387V) *de novo*
**TUBB3**											
P17816 ^§^	F, 4 yrs	no	no	/	NO	NA	NA	NA	/	no MCD	c.1228G > A (p.E410K) *de novo*
105814 ^§^	M, 4 yrs 9 mo	no	no	/	Irregular Slowing	0	Irregular Asym Asynchronous	SA Right PO and diffuse	/	no MCD	c.862G > A (p.E288K) *de novo*
P120818	M, 34 yrs	no	no	/	NA	NA	NA	NA	/	No MCD	c.689C > T (p.S230L)
P109418	M, 12 yrs	no	no	/	Irregular Slowing	SA PO bilateral	Irregular Asynchronous	SA paroxysmal PO bilateral	/	Perisylvian PMG	c.728C > T (p.P243L)

AEDs: Antiepileptic drugs; Asym: asymmetric; BA: Background activity; BFA: burst of fast activity; C: central; cn: cranic nerve; EA: Epileptiform Abnormalities; F: female; FS: focal seizures; ISL: intermittent light stimulation; L: Left; LTG: Lamotrigine; M: male; MCDs: malformations of cortical development; mo: months; NA: not available; NEM: not-epileptic myoclonus; no: not observed; O: occipital; P: parietal; PMG: Polymicrogyria; PMG-multi: multifocal Polymicrogyria; R: right; S: Sleep; SCH: Schizencephaly; Symp_Gyr: simplified gyral pattern with focal polymicrogyria: SA: slow abnormalities, SE: status epilepticus; T: temporal; VPA: Valproic acid; W: Wakefulness; yrs: years. ^§^ Patients already reported (Romaniello, Arrigoni, Panzeri et al., Eur Radiol (2017) 27:5080-5092.

## Supplementary Materials

The following are available online at https://www.mdpi.com/2073-4409/8/7/669/s1.
