# Supplementary material for: Epilepsy in Tubulinopathy: Personal Series and Literature Review
Source: Cells. 2019 Jul 2;8(7):669. doi: 10.3390/cells8070669 (PMC6678821; doi:10.3390/cells8070669)
Supplement: Supplementary file 1 [file cells-08-00669-s001.pdf]

Gene List of the targeted NGS MCD and CCA gene panel

AKT3,ALX1,ALX3,ALX4,AMPD2,ARFGEF2,ARID1B,ARX,ASPM,ATR,ATRX,B3GALTL,BRPF1,c12orf57,C6orf70,CASK,CCND2,CDK5RAP2,CDON,CENPJ,CEP170,CHMP1A,COL4A1,CREBBP,CYP11A1,DCHS1,DCLK1,DCX,DHCR24,DHCR7,DIS3L2,DISC1,DISP1,DLL1,DMRTA2,DYNC1H1,DYRK1A,EARS2,EFNB1,EMX1,EOMES,EP300,ERBB4,ERMARD,EXOSC3,FAM36A,FGF8,FGFR1,FGFR2,FLNA,FOXC1,FOXG1,FOXH1,FZD10,GLI2,GLI3,GPR56,GPSM2,HCCS,HESX1,HNRNPU,IGBP1,IGFBP1,ISPD,ITPA,KAL1,KAT6B,KATNB1,KIAA1279,KIF14,KIF1A,KIF1B,KIF21A,KIF2A,KIF5C,KIF7,L1CAM,LAMB1,LAMC3,LRP2,MCPH1,MED12,MID1,NDE1,NFIB,NPC1,NR2F1,NSD1,NTRK1,NTRK3,OCEL1,OPA1,OTX2,PAFAH1B1,PAX6,PEX1,PHF10,PIK3R2,POLR3A,POLR3B,POMT1,POMT2,PTCH1,PTPRS,PYCR1,RAB3GAP1,RARS2,RELN,RFX3,ROBO1,ROBO3,RPS6KA3,RTTN,SATB2,SEPSECS,SHH,SIX3,SLC12A6,SOX2,SPOCK1,SRPX2,TBCD,TBCE,TCF4,TDGF1,TEAD1,THBS2,TMEM5,TSC1,TSC2,TSEN15,TSEN2,TSEN34,TSEN54,TUBA1A,TUBA8,TUBB,TUBB2A,TUBB2B,TUBB3,TUBB4A,TUBG1,VAX1,VRK1,WDR47,WDR62,ZBTB18,ZEB2,ZIC2.

Gene List of the targeted NGS epilepsy gene panel

AARS, ADGRV1, ADRA2B, ADSL, ALDH4A1, ALDH7A1, ALG13, ALPL, ARHGEF15, ARHGEF9, ARX, ASAH1, ATP1A2, ATP1A3, BRD2, CACNA1A, CACNA1H, CACNA2D2, CACNB4, CBL, CDKL5, CERS1, CHD2, CHRNA2, CHRNA4, CHRNB2, CLCN2, CLCN4, CLN8, CLTC, CNKSR2, CNTNAP2, CPA6, CPLX1, CSNK1G1, CSNK2B, CTNND2, DEPDC5, DHDDS, DNM1, DOCK7, DYNC1H1, EEF1A2, EFHC1, EIF2S3, EMC1, EPM2A, FASN, FLNA, FOXG1, GABBR2, GABRA1, GABRA2, GABRA3, GABRB2, GABRB3, GABRD, GABRG2, GAL, GNAO1, GOSR2, GRIA1, GRIN1, GRIN2A, GRIN2B, HCN1, HCN4, HDAC4, HNRNPU, IDH3A, IQSEC2, JRK, KCNA1, KCNA2, KCNB1, KCNC1, KCND2, KCND3, KCNH1, KCNH5, KCNJ10, KCNMA1, KCNQ2, KCNQ3, KCNT1, KCTD7, KPNA7, KPTN, LGI1, LMNB2, MBD5, MDH2, MEF2C, MFSD8, MICAL1, MTOR, NACC1, NAPB, NECAP1, NEDD4L, NHLRC2, NPRL2, NPRL3, NRXN1, NTRK2, NUS1, OTUD7A, PCDH19, PIGA, PIGC, PIGN, PIGO, PIGP, PIGQ, PIGV, PLCB1, PNKP, PNPO, POLG, PRIMA1, PRRT2, PTPN23, PURA, QARS, RAB11A, RBFOX1, RBFOX3, RHOBTB2, ROGDI, RYR3, SCARB2, SCN10A, SCN1A, SCN1B, SCN2A, SCN8A, SCN9A, SEPSECS, SETD2, SIK1, SLC12A5, SLC13A5, SLC25A22, SLC2A1, SLC35A2, SLC6A1, SLC6A9, SMC1A, SMS, SNAP25, SPTAN1, SRPX2, ST3GAL3, STX1B, STXBP1, SYN1, SYNGAP1, SZT2, TBC1D24, TBCD, TCF4, TNK2, TPP1, TRAK1, UBA5, UNC80, WDR45, WWOX, YWHAG, ZDHHC9.

Table 1S. Epilepsy in *TUBA1A*, *TUBB2B* and *TUBB3* genes mutations

Reference	N °of Epileptic Patients/ Total	MCDs on MRI	Seizure type	Age at onset	EEG findings	Response to AEDs
**TUBA1A**	**44/155**					
Keays et al, 2007	1/2					
	K1	Lissencephaly	TCS	2 yrs	NA	Refractory
Poirier et al, 2007	3/8					
	P3	Posterior Agyria	TCS	Early	NA	Refractory
	P5	SBH	TS	NA	NA	Controlled
	P8	Posterior Pachygyria	TCS	Early	NA	Refractory
Bahi-Buisson et al, 2008	2/6					
	BB2	Perysilvian Pachygyria	TS	Early	NA	Refractory
	BB6	Posterior Pachygyria	Spasms	Early	NA	Controlled (VPA)
Morris-Rosendahl et al, 2008	5/5					
	MR1	Agyria-pachygyria P>A	FS and TCS	15 mo	NA	NA
	MR2	Predominantly agyria P>A	NA	9 mo	NA	NA
	MR3	Agyria-pachygyria P>A	FS	8 mo	NA	NA
	MR4	Pachygiria with SBH	FS (motor)	5 mo	NA	NA
	MR5	Pachygiria with SBH	Generalized (not specified)	4 weeks	NA	NA
Kumar et al, 2010	0/17					
Jansen et al, 2011	2/4					
	J1	Agyria-Pachygyria A>P	FS	Birth	NA	Refractory
	J2	Perysilvian PMG	FS	5 mo	NA	Refractory
Sohal et al, 2012	1/1					
	S1	Lissencephaly	Spasms (tonic, trunk and limbs)	10 weeks	NA	Refractory
Mokanszki et al, 2012	1/1					
	M2	Agyria-pachygyria P>A	Spasms	5 mo	interictal: generalized spike-waves, and bursts in sleep	Controlled (VGB)
Cushion et al, 2013	0/2					
Okumura et al, 2013	1/1					
	O1	Thin Cortex	Spasms; TS	8 mo; 19 mo	NA	Refractory
Poirier et al, 2013	2/3					
	P1	Perysilvian PMG	Focal SE, FS	2,5 yrs	NA	Refractory
	P2	Perysilvian PMG	FS	3 mo	NA	Refractory
Zanni et al, 2013	0/1					

Bahi-Buisson et al, 2014	0/13					
Hikita et al, 2014	1/1					
	H1	Agyria	Generalized (not specified)	9 days	NA	Controlled
Kamiya et al, 2014	1/1					
	K1	Lissencephaly	Generalized (not specified)	1 yr	NA	Refractory
Shimojima et al, 2014	0/1					
Myers et al, 2015	1/1					
	M1	Mild Posterior Simplified cerebral gyral pattern	FS, Spasms, Polymorphic	2 mo	NA	Partially controlled (KD and IM therapy)
Oegema et al, 2015	2/2					
	O1	Diffuse irregular gyration and sulcation	Absences	NA	NA	NA
	O2	Diffuse irregular (L>R) gyration and sulcation	Spasms	NA	NA	Refractory
Yokoi et al, 2015	2/2					
	Y1	Extremely thin cerebral parenchyma	FS	Birth	ictal: extremely poor BA, focal rhythmic delta waves	Partially controlled (PB)
	Y2	Lissencephaly	NA	8 months	NA	Controlled (PB, ZNS)
Bamba et al, 2016	2/2					
	B1	Lissencephaly	Generalized (not specified)	Early	NA	Refractory
	B2	Agyria-pachygyria P>A	Generalized (not specified)	Early	NA	Refractory
Mencarelli et al, 2017	1/1					
	M1	Cortical Dysgenesis	FS	1 mo	interictal: irregular BA, slow waves posteriorly on the left side	NA
Romaniello et al, 2017	6/14					
	R1	PMG-multi	FS	21 days	See Table 1	Controlled (LEV)
	R2	Simp_Gyr_occipital	FS	NA	NA	NA
	R3	no MCDs	Spasms	NA	NA	NA
	R4	no MCDs	Spasms, TCS	NA	NA	Refractory
	R5	Perysilvian-PMG	Myoclonic	NA	NA	NA
	R6	No MCDs	Myoclonic, focal SE, FS	3 mo	See Table 1	Controlled (VPA, ETS)
Gardner et al, 2018	2/3					
	G1	Perysilvian PMG	Focal SE	3 yrs	ictal: subclinical left occipital lobe seizures	Controlled (OXC)
	G2	No MCDs	Absences and TCS	1 yrs	NA	NA
Sato et al, 2018	1/1					

	S1	Poroencephaly, occipital PMG	TCS	7 mo	interictal: spikes in the right frontal lobe	Partially controlled (PB, CBZ, LEV)
Hebebrand et al, 2019	2/3					
	H1	Dysgyria<br>NA	FS, Spasms	NA	NA	NA
	H2		Generalized (not specified)	NA	NA	NA
Hebebrand, from databases °	4/58					
		NA	NA	NA	NA	NA
Unpublished patient (our series)	1/1<br>R1	Perisylvian dysgyria	Spasms, FS	18 mo, 3 yrs	See Table 1	Controlled (ACTH, VPA)
**TUBB2B**	**19/39**					
Jaglin et al, 2009	3/4					
	J2	Frontal and temporal lobes PMG	Spasms	3 mo	NA	NA
	J3	Asymmetric (L frontal, parietal and temporal lobes) PMG	Generalized (not specified)	NA	NA	Occasional
	J4	Asymmetric (L frontal, parietal and temporal lobes) PMG	Generalized (not specified)	NA	NA	NA
Cederquist et al, 2012	0/3					
Guerrini et al, 2012	1/3					
	G1	Diffuse PMG more severe in perysilvian regions	TS; atypical absences	11 mo; 3 yrs	interictal: ESES	NA
Cushion et al, 2013	4/4					
	C1	Bilateral Asymmetric PSPMGL L frontal and parietal lobes	NA	6 mo	NA	NA
	C2	Bilateral Symmetric PSPMGL medial temporal lobes	FS and Generalized (not specified)	11 mo	NA	NA
	C3	Bilateral symmetric Agyria and thick, irregular subcortical and of grey matter	TCS, MS, TS	4 mo	NA	NA
	C4	Bilateral Symmetric DPMGL	NA	3 mo	NA	NA
Amrom et al, 2014	2/3					
	A1	Asymmetric (R perysilvian	FS (nocturnal)	NA	NA	NA

		region) PMG				
	A2	Asymmetric (R FP and L frontal lobes) PMG	Spasms	NA	NA	NA
Bahi-Buisson et al, 2014	0/8					
Jamuar et al, 2014	0/1					
Oegema et al, 2015	0/2					
Breuss et al, 2017	0/1					
Geiger et al, 2017	1/1					
	G1	Temporal-Parietal pachygyria	TCS	30yrs	NA	Controlled (LEV)
Romaniello et al, 2017	7/8					
	R1	Generalized PMG+ SCH	Spasms, FS	5 mo	See Table 1	Controlled (VPA, LTG)
	R2	Generalized PMG	FS	Birth	NA	NA
	R3	Simp_Gyr_occipital	NA	NA	NA	NA
	R4	Generalized PMG+ SCH	Spasms, FS	18 mo	See Table 1	Partially controlled (VPA)
	R5	Symp_Gyr, periventricular heterotopia, subcortical linear heterotopia, small temporal lobes	Spasms, FS	7 mo; 12 mo	See Table 1	Controlled (ACTH, PB, CBZ)
	R6	Perysilvian-PMG	NA	NA	NA	NA
	R7	No MCDs	FS	NA	NA	Controlled (LEV)
Jimenez et al, 2019	1/1					
	J1	Opercular dysgyria	TS	2yrs	NA	Controlled (VPA, LEV)
**TUBB3**	**3/62**					
Poirier et al, 2010	2/9					
	P1	Global GD	NA	neonatal	NA	Occasional
	P2	Perysilvian PMG	Prolonged febrile seizures	NA	NA	NA
Tischfield et al, 2010	0/29					
Chew et al, 2013	0/2					
Bahi-Buisson et al, 2014	0/1					
MacKinnon et al, 2014	0/4					
Oegema et al, 2015	1/3					
	O1	Diffuse irregular gyration and sulcation, multiple shallow sulci	NA	28 mo	NA	Partially controlled by AEDs
Fukumura et al, 2016	0/1					
Whitman et al, 2016	0/4					
Shimojima et al, 2016	0/1					

Patel et al, 2017	0/1
Romaniello et al, 2017	0/6
Nakamura et al, 2018	0/1

§Early: < 12 months;

°mutations found in public databases, reported by Hebebrand M et al, 2019

ACTH: adrenocorticotropic hormone; AEDs: antiepileptic drugs; A>P: posterior-to- anterior gradient of agyria-pachygyria BA: background activity; CBZ: carbamazepine; DPMGL: diffuse PMG-like; ESES: electrical status epilepticus during sleep; ETS: Ethosuccimide; FP: fronto-parietal; FS: focal seizures; GD: gyral disorganization; IM: intravenous immunoglobulin; KD: ketogenic diet; L: left; LEV: levetiracetam; LTG: lamotrigine; MCDs: malformations of cortical development; mo: months; MS: myoclonic seizures; NA: not available; OXC: oxcarbazepine; P>A: posterior-to- anterior gradient of agyria-pachygyria; PB: phenobarbital; PMG: polymicrogyria; PMG-multi: multifocal polymicrogyria; PSPMGL: perysilvian PMG-like; R: right; SBH: subcortical band heterotopia; SCH: schizencephaly; SE: status epilepticus; TCS: tonic-clonic seizures; TS: tonic seizures; yrs: years; VPA: valproic acid; ZNS: zonisamide.

Table 2S. Epileptological findings in TUBA1A, TUBB2B and TUBB3 genes mutations

	**TUBA1A** (n=44/155) 28%		**TUBB2B** (n=19/39) 49%		**TUBB3** (n=3/62) 5%	
**Seizures***	6 case NA		4 cases NA		2 cases NA	
**Focal**	15	39%	7	47 %	/	
**Generalized**					/	
**Not specified**	1	3 %	3	20 %		
**Absence**	2	6 %	1	7 %		
**TCS**	7	18 %	2	13%		
**TS**	3	8%	2	13%		
**MS**	2	6%	1	7 %		
**Spasms**	10	26 %	5	33%	/	
**Polymorphic**	1	3 %	/		/	
**Status epilepticus**	3 (focal)*	8 %	/		/	
**Febrile**	/		/		1	100 %
**Range of age**	13 cases NA		7 cases NA		1 case NA	
	neonatal-3 years		3 months- 3 years		neonatal-28 months	
**EEG§**	36 cases NA		15 cases NA		3 NA	
**Ictal**	extremely poor BA, focal rhythmic delta waves 1 case)<br>subclinical left occipital lobe seizures (1 case)		/		/	
**Interictal**	irregular BA, slow waves posteriorly on the left side (1 case)<br>spikes in the right frontal lobe (1 case)		ESES (1 case)		/	
**Response to AEDs**	17 cases NA		12 cases NA		1 case NA	
**Controlled**	9	33%	5	72%	/	
**Occasional**	/		1	14%	1	50 %
**Partially controlled**	3	11%	1	14%	1	50 %
**Refractory**	15	56%	/		/	

AEDs: antiepileptic drugs; BA: background activity; ESES: electrical status epilepticus during sleep; NA: not available; *in same patients more types of seizures occur (see Table 1S); §EEG of our series are described in Table 1

Supplementary References

Cederquist GY, Luchniak A, Tischfield MA, Peeva M, Song Y, et al. An inherited TUBB2B mutation alters a kinesin-binding site and causes polymicrogyria, CFEOM and axon dysinnervation. Hum Mol Genet 2012, 21:5484-5499.

Chew S, Balasubramanian R, Chan WM, Kang PB, Andrews C, et al. A novel syndrome caused by the E410K amino acid substitution in the neuronal β-tubulin isotype 3. Brain 2013, 136:522-535.

Fukumura S, Kato M, Kawamura K, Tsuzuki A, Tsutsumi H. A Mutation in the Tubulin-Encoding TUBB3 Gene Causes Complex Cortical Malformations and Unilateral Hypohidrosis. Child Neurol Open Sep 1;3:2329048X16665758. doi: 10.1177/2329048X16665758. eCollection 2016 Jan-Dec.

Geiger JT, Schindler AB, Blauwendraat C, Singer HS, Scholz SW. TUBB2B Mutation in an Adult Patient with Myoclonus-Dystonia. Case Rep Neurol 2017, 9:216-221.

Jamuar SS, Lam AT, Kircher M, D'Gama AM, Wang J, et al. Somatic mutations in cerebral cortical malformations. N Engl J Med 2014, 371:733-743.

Jimenez J, Herrera DA, Vargas SA, Montoya J, Castillo M. β-Tubulinopathy caused by a mutation of the TUBB2B gene: magnetic resonance imaging findings of the brain. Neuroradiol J 2019, 32:148-150.

MacKinnon S, Oystreck DT, Andrews C, Chan WM, Hunter DG, Engle EC. Diagnostic distinctions and genetic analysis of patients diagnosed with moebius syndrome. Ophthalmology 2014:121:1461-1468.

Nakamura Y, Matsumoto H, Zaha K, Uematsu K, Nonoyama S. TUBB3 E410K syndrome with osteoporosis and cough syncope in a patient previously diagnosed with atypical Moebius syndrome. Brain Dev 2018:40:233-237.

Patel RM, Liu D, Gonzaga-Jauregui C, Jhangiani S, Lu JT, et al. An exome sequencing study of Moebius syndrome including atypical cases reveals an individual with CFEOM3A and a TUBB3 mutation. Cold Spring Harb Mol Case Stud 2017 Mar;3(2):a000984. doi: 10.1101/mcs.a000984.

Shimojima K, Okamoto N, Yamamoto T. A novel TUBB3 mutation in a sporadic patient with asymmetric cortical dysplasia. Am J Med Genet A 2016:170:1076-1079.
